# Cartesian Control of Sit-to-Stand Motion Using Head Position Feedback

**DOI:** 10.1155/2020/1979342

**Published:** 2020-08-20

**Authors:** Samina Rafique, M. Najam-ul-Islam, M. Shafique, A. Mahmood

**Affiliations:** ^1^Electrical Engineering Department, Bahria University, Islamabad 44230, Pakistan; ^2^Biomedical Engineering Department, Riphah International University, Islamabad 44230, Pakistan

## Abstract

Sit-to-stand (STS) motion is an indicator of an individual's physical independence and well-being. Determination of various variables that contribute to the execution and control of STS motion is an active area of research. In this study, we evaluate the clinical hypothesis that besides numerous other factors, the central nervous system (CNS) controls STS motion by tracking a prelearned head position trajectory. Motivated by the evidence for a task-oriented encoding of motion by the CNS, we adopt a robotic approach for the synthesis of STS motion and propose this scheme as a solution to this hypothesis. We propose an analytical biomechanical human CNS modeling framework where the head position trajectory defines the high-level task control variable. The motion control is divided into low-level task generation and motor execution phases. We model CNS as STS controller and its Estimator subsystem plans joint trajectories to perform the low-level task. The motor execution is done through the Cartesian controller subsystem that generates torque commands to the joints. We do extensive motion and force capture experiments on human subjects to validate our analytical modeling scheme. We first scale our biomechanical model to match the anthropometry of the subjects. We do dynamic motion reconstruction through the control of simulated custom human CNS models to follow the captured head position trajectories in real time. We perform kinematic and kinetic analyses and comparison of experimental and simulated motions. For head position trajectories, root mean square (RMS) errors are 0.0118 m in horizontal and 0.0315 m in vertical directions. Errors in angle estimates are 0.55 rad, 0.93 rad, 0.59 rad, and 0.0442 rad for ankle, knee, hip, and head orientation, respectively. RMS error of ground reaction force (GRF) is 50.26 N, and the correlation between ground reaction torque and the support moment is 0.72. Low errors in our results validate (1) the reliability of motion/force capture methods and anthropometric technique for customization of human models and (2) high-level task control framework and human CNS modeling as a solution to the hypothesis. Accurate modeling and detailed understanding of human motion can have significant scope in the fields of rehabilitation, humanoid robotics, and virtual characters' motion planning based on high-level task control schemes.

## 1. Introduction

Sit-to-stand (STS) movement is a skill that helps determine the functional level of a person. The ability to rise from sitting to standing is critical to a person's quality of life, as it is linked with the functional independence of an individual. Studies on the hierarchy of disability indicate that problem in STS starts at a later stage than problems in walking commence [1]. Biomechanical literature is replete with studies aimed at determining various variables that contribute to STS motion. Kinematic variables like joint positions, velocities, acceleration, Centre of Mass (CoM), Centre of Gravity (CoG), and Center of Pressure (CoP) and kinetic variables like ground reaction forces (GRF), joint torques, and ground reaction torques play an important role as feedback elements in STS motion control [2].

Of all sensory inputs, head position and orientation too are the area of researchers' interest. There is ample clinical evidence that head position feedback to CNS plays a role in the control of human motion and maintenance of balance. The study [3] shows that human motion control and maintenance of balance by CNS rely on inputs from vision, proprioception, tactile/somatosensory, and vestibular systems. The multisensory integration, combined with motion control, undergoes both quick and slow alterations which are termed as fast and slow dynamics in CNS, respectively. For any voluntary motion, CNS anticipates set patterns of inputs from multisensory systems. Vestibular sense, in conjunction with neck proprioception, estimates body orientation. The vestibular system senses linear and angular head motion, and the CNS uses this information for posture and gaze control [4]. A study in [5] suggested that visual perception played a role in balance control during STS. The role of head position feedback to CNS in smooth execution of STS is also studied in [6], and the dependence of the STS movement on the Centre of Mass (CoM) and head positions during the task is analyzed.

To evaluate clinical hypotheses, computer simulations act as a powerful tool. Human motion can be synthesized and analyzed in a simulation environment. Like all other motions, the behavioral richness exhibited in natural human STS transfer results from a complex interplay of biomechanical and neurological factors [7]. An adequate understanding of these factors is a prerequisite to understanding the overall mechanism of human STS motion as well as providing a means for its synthesis. In a broader sense, basic constituents of the human motor system include biomechanical plants and CNS. Based on some specified task, CNS performs motor planning which culminates low-level control issued as a motor command to biomechanical plant. Some knowledge of biomechanical plant is also assumed to be encoded in CNS. Typically, CNS is modeled to serve only a limited function. One possible model which is the most commonly used is the joint space control. It is possible to divide motion control into the task generation phase and a motor execution phase. This abstraction is more relevant to the design of engineered systems that augment physiological systems. Synthesis of human motion involves accurate reconstruction of movement sequences, modeling of musculoskeletal kinematics, dynamics, and actuation of segment joints [8]. Task-based methods used in robotics may be leveraged to provide novel musculoskeletal modeling methods and physiologically accurate performance predictions. Advantage of robotic-based effort models frequently utilizes quantities that are derivable purely from skeleton kinematics and that are not specific to muscle actuation. Since the evaluation of a system can be only as good as the model itself, the accuracy of the results primarily depends upon the quality of the human biomechanical model. Human STS is performed almost entirely in the sagittal plane [9]. Typically, human biomechanical models comprise a multilevel inverted pendulum, whose motion is governed by Euler-Lagrange equations. For motion analysis and development of a control scheme, usually, an analytical model based on general physical parameters is realized. Such models and control schemes are extensively available in the literature on motion analysis [10, 11] and the design of robotic devices [12]. To analyze a motion mechanism more accurately, the simulated motion must be compared with actual human motion. For this purpose, custom biomechanical models are developed that are more useful in the design and tuning of customizable motion assistance and rehabilitation devices. Custom human biomechanical models are based upon Body Segment Parameter (BSP) values. Reimer et al. [13] have given an overview of methods available for the estimation of BSP. Weighing coefficient-based methods are convenient but the error in results can be up to 40%. Geometric approaches are good (error less than 5%), but tedious as the number of body part measurements can go even higher than 240. Medical imaging is also accurate (error < 5%) but needs expensive equipment in addition to dangerous exposure to radiation. Among all these methods, marker-based motion capture system was reported as the accurate one, despite its limitations in terms of cost, the need for a controlled environment, high sensitivity to noise, line of sight capture, etc.

To validate the modeling technique, the simulated motion profiles are compared with experimental results. In [12], the proposed 6-link human model was checked for its accuracy using references from experimental data. The relation of two kinetic variables, GRF and reaction moments, was recorded from subjects and compared with the same forces resulting in simulations [14]. The regression plots of two variables endorsed similarity between them during the gait cycle. The validation of the modeling scheme through the experimental result is also done in [8, 15, 16]. In [17], the researcher collected data of STS motion using infrared cameras and force plates and applied the data to a multisegment biomechanical model for the analysis of the kinematic contribution of major body segments.

Synthesis of human-like motion finds its application both in simulation and physical settings: in computer graphics, this leads to autonomously generating realistic motion for virtual characters. The intent is to direct these virtual characters using high-level task for which low-level motion control is automatically generated. Similarly, the robotics community seeks a high-level control framework for robotic systems [7].

In this study, we evaluate the clinical hypothesis that besides numerous other factors, CNS controls STS motion by tracking a prelearned head position trajectory. Motivated by the evidence [7] for a task-oriented encoding of motion by the CNS, we adopt a robotic approach for the synthesis of STS motion and propose this scheme as a solution to this hypothesis. We propose an analytical biomechanical human CNS modeling framework where the head position trajectory defines the high-level task control variable. We do extensive motion and force capture experiments on human subjects to validate our analytical modeling scheme. To the best of our knowledge, this is the first study of STS motion and force capture in the sagittal plane (2D). We used marker-based optical motion capture system and force plate (1) to collect kinematic and kinetic data during this voluntary motion and (2) to realize a custom human biomechanical model in the sagittal plane as close as possible to real human beings. We first scale our biomechanical model to match the BSP values of the subjects. We do dynamic motion reconstruction through the control of the simulated custom human CNS models to follow the captured head position trajectories in real time. This study is the part of an ongoing study that is aimed at determining different variables involved in human STS motion. The previous work [11, 18, 19] comprised the analytical approach, and this work is based on experimental analysis of STS motion. This paper is organized as follows: first, we provide the details of the analytical modeling framework for STS motion synthesis followed by the experimental setup and data collection of STS motion on human subjects. Next, we discuss the human biomechanical model scaling for custom human models. We simulate each subject's STS motion and compare them with experimental results. Finally, we discuss the validity of the proposed design methodology for its physiological relevance to the STS maneuver.

## 2. Materials and Methods

**Figure 1:**
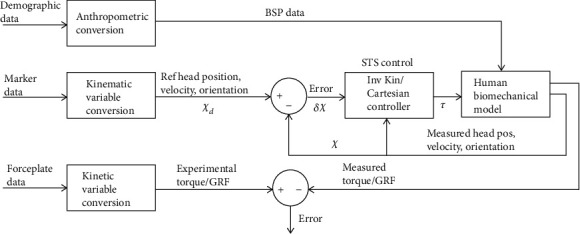
Workflow of STS motion control scheme.

We design a biomechanical human CNS model (as shown in Figure 1) to synthesize and control STS motion by tracking only head trajectory *𝒳*_*d*_ as a reference and head position *𝒳* as the only measurement. Since the reliability of the motion control is primarily linked with the accuracy of the human biomechanical model, we compare experimental and simulated forces and fine-tune the model to reduce the error to a minimum. Hence, force measurement does not play a role in motion synthesis or control, it is meant only for modeling scheme validation.

### 2.1. The Analytical Modeling Framework for STS Motion Synthesis

We develop an analytical human CNS modeling framework to generate STS motion. Our modeling scheme comprises the following steps:

- A general four-segment human biomechanical model in the sagittal plane based on BSP from the literature [2, 9–11, 19–21] is realized in SimMechanics
- We analytically generate head trajectory [22] to be used as the reference
- We design the STS controller to emulate human CNS, capable of (a) estimating joint angles using inverse kinematics based on head position measurements *𝒳* and (b) generating joint actuation torque commands *τ* by Cartesian control based on head position error *δ𝒳*

### 2.2. Experimental Validation of Modeling Scheme

- The physical parameter data collected from 7 subjects are converted into BSP using the weighing coefficient method of anthropometry. BSP values are used to realize custom/subject-specific human biomechanical models
- We capture motion and force data of STS maneuver from subjects using multiple infrared cameras and passive reflective markers. We extract custom head trajectories from motion data and torques and ground reaction forces (GRFs) from force data
- STS motion is reconstructed for each custom human CNS model. Custom head trajectories are used as the reference for respective models. Simulated motions are analyzed and compared with experimental motion. The materials and methods section should contain sufficient detail so that all procedures can be repeated. It may be divided into headed subsections if several methods are described

## 3. Analytical Modeling Framework

### 3.1. The General Human Biomechanical Model

**Figure 2:**
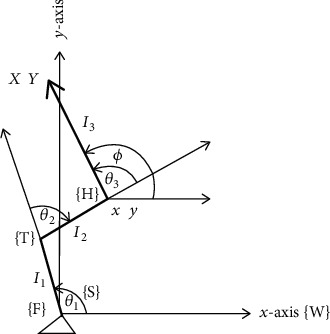
Three DoF biomechanical human model defined in the body frame. {S}, {T}, and {H} represent shank, thigh, and HAT frames for segments *l*_1_, *l*_2_, and *l*_3_.

A general four-link rigid body human model (as shown in Figure 2) is used to simulate STS motion. The physiological parameters of the model (as shown in Table 1) have been borrowed from literature including our previous work [11, 18, 19, 21].

The model has three degrees of freedom (DoF). Four links include the foot, shank, thigh, and the upper body, which we termed as a single link called Head-Arm-Trunk (HAT). A triangular base of support represents the foot fixed on the ground. Since the key movements of joints and limbs during STS take place in the sagittal plane only, we limit our model to planar two-dimensional (2D) motions (in the Cartesian plane). All joints are revolute (hinge-like), and the model is an open-chain mechanism with three actuators at each joint. *θ*_1_, *θ*_2_, and *θ*_3_ represent ankle, knee, and hip joint positions, respectively. We refer the shank, thigh, and HAT as links *l*_1_, *l*_2_, and *l*_3_, respectively. (*X*, *Y*) is the head position, and (*x*, *y*) is the hip position in Cartesian coordinates. ∅ is the head orientation in the World frame {W}.

### 3.2. Analytical Reference Trajectory

The model tracks a generalized head position trajectory, generated analytically using an unforced state-space system borrowed from [22], and modified accordingly.

### 3.3. CNS Modeling: STS Controller Design

The CNS is modeled as STS controller comprising two subsystems: an Estimator and a Cartesian controller.

#### 3.3.1. Estimator

The estimation of joint angles is based on the inverse kinematics of the human biomechanical model.

*(1) Forward Kinematics (FK) Analysis*. Forward kinematics maps joint space (*θ*_*n*_) into Cartesian space (*x*, *y*, *ϕ*) [23], where *ϕ* is the orientation of a point in the Cartesian plane with respect to the World reference {W}. To determine the head position (*X*, *Y*), the set of kinematic equations is given as

(1) $$\begin{array}{c} X=l_{1}c_{1}+l_{2}c_{12}+l_{3}c_{123}, \end{array}$$

(2) $$\begin{array}{c} Y=l_{1}s_{1}+l_{2}s_{12}+l_{3}s_{123}, \end{array}$$

where *c*_1_ stands for cos(*θ*_1_), *c*_12_for cos(*θ*_1_ + *θ*_2_), *s*_1_ for sin(*θ*_1_), and so on. Also,

(3) $$\begin{array}{c} \phi =\theta_{1}+\theta_{2}+\theta_{3}, \end{array}$$

where *ϕ* is the orientation of HAT (or head) with respect to the *x*-axis. The generalized coordinate is a compact notation *p* = [*X*, *Y*, *ϕ*].

*(2) Inverse Kinematics (IK) Analysis*. To estimate joint angles, the IK problem is solved. First, *p* is used to find a unique hip position (*x*, *y*) to reduce the problem at hand from four to three links. To find hip position (*x*, *y*), hip joint angle constraint, i.e., 0 ≤ *θ*_3_ ≤ *π* is imposed. The solution then simplifies

(4) $$\begin{array}{c} x=X+l_{3}\text{c}\left(\pi -\phi \right), \end{array}$$

(5) $$\begin{array}{c} y=Y-l_{3}\text{s}\left(\pi -\phi \right). \end{array}$$

Using algebraic manipulation, the three joint angles inferred from head position are

(6) $$\begin{array}{c} \theta_{2}=\text{atan}2\left(s_{2},c_{2}\right), \end{array}$$

(7) $$\begin{array}{c} \theta_{1}=\text{atan}2\left(y,x\right)-\text{atan}2\left(l_{2}s_{2},\left(l_{1}+l_{2}c_{2}\right)\right), \end{array}$$

(8) $$\begin{array}{c} \theta_{1}+\theta_{2}+\theta_{3}=\text{atan}2\left(s\phi ,c\phi \right)=\phi , \end{array}$$

where atan2 is the MATLAB command for four-quadrant tan^−1^ with arguments in bracket representing vertical and horizontal components of the position vector.

#### 3.3.2. Cartesian Control

Cartesian control refers to the position control of the head, following a required trajectory in Cartesian space.

*(1) Equation of Motion*. Dynamic equation of motion of the human biomechanical model in joint space is given by

(9) $$\begin{array}{c} \tau =M\left(\theta \right)\ddot{\theta }+V\left(\theta ,\dot{\theta }\right)+G\left(\theta \right), \end{array}$$

where $\ddot{\theta }$, $\;\dot{\theta }\;$, and *θ* are *n* × 1 joint angular acceleration, velocity, and position vectors, respectively. *M*(*θ*) is the *n* × *n* inertia matrix of the model, $V\left(\theta ,\dot{\theta }\right)$ is *n* × 1 vector of centrifugal and Coriolis terms, *G*(*θ*) is the *n* × 1 vector of gravity terms, and *τ* is the *n* × 1 torque vector. Modifying dynamic equation from joint space to Cartesian space [23],

(10) $$\begin{array}{c} F=M_{x}\left(\theta \right)\ddot{X}+V_{x}\left(\dot{\theta },\theta \right)+G_{x}\left(\theta \right), \end{array}$$

where *ℱ* is the appropriate force-torque vector, and *𝒳* is the position and orientation of the head in Cartesian space. *M*_*x*_(*θ*) is the mass-inertia matrix in Cartesian space and so on.

A trajectory conversion process thus required

(11) $$\begin{array}{c} \theta_{d}=\text{inv kin }\left(X_{d}\right), \end{array}$$

where *𝒳*_*d*_ is the desired head position trajectory in Cartesian space, and *θ*_*d*_ is the vector of corresponding joint angles. The inv kin operator refers to the inverse kinematic procedure used for the inference of joint angles from the position of the end effector.

*(2) Transpose Jacobian Control*. In this scheme, measured position *𝒳* is compared to desired position *𝒳*_*d*_ to form an error *δ𝒳* in Cartesian space. The error vector is then applied to control law to compute the Cartesian force vector, *ℱ*, which is that fictitious force if applied at the head will tend to reduce Cartesian error. The Cartesian force vector is then mapped into joint torque vector *τ* using transpose Jacobian conversion.

*(3) The Velocity of the Head*. Description of angular velocity *ω* of link *i* + 1 with respect to respective frame is given by

(12) ωi+1i+1=Rii+1ωii+θ˙i+1Z^i+1i+1,

where *i* = 0,1,2 refers to link number, *R* is a rotation matrix, and *Z* is the axis of joint rotation. The linear velocity *v* is given by

**Figure 3:**
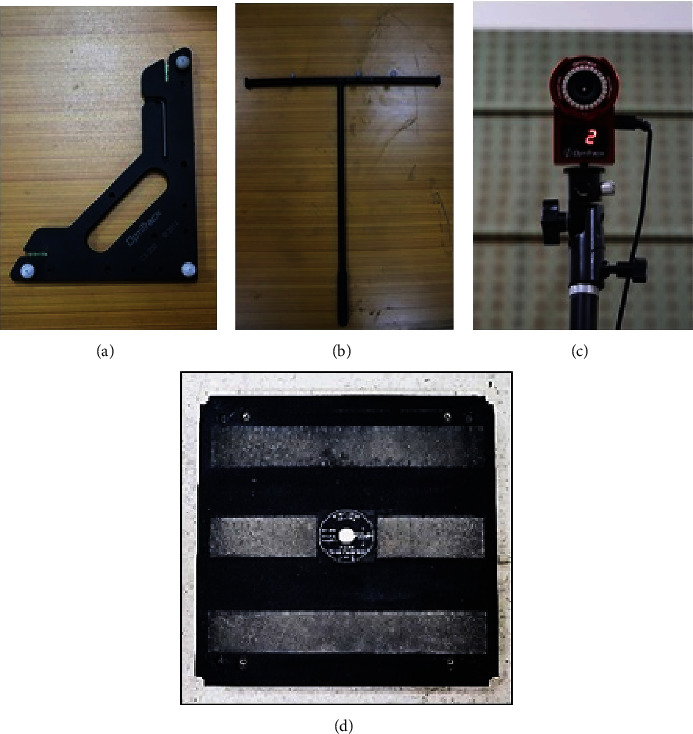
Motion capture equipment by OptiTrack. (a) Calibration square, (b) calibration wand, (c) infrared camera, and (d) the Pasco force plate for force data capture.

(13) vi+1i+1=Rii+1vii+ωii×Pi+1i,

where *P* is the head position vector. For the model shown in Figure 3, the angular and linear velocity components of the head in three axes are given, respectively:

(14) −ω3=3ω2=00θ˙1+θ˙22,

(15) v3=l1s2θ˙1l1c2θ˙1+l2θ˙1+θ˙203.

To find these velocities with respect to fixed foot-shank frame {F}, using the rotation matrix

(16) R=30R30R21R32=c12−s120s12c120001,

(17) v3=R30v330=−l1s1θ˙1−l2s12θ˙1+θ˙2l1c1θ˙1+l2c12θ1+θ˙20.

*(4) The Jacobian*. Jacobian is a nonlinear time-varying matrix that relates joint angular velocities to linear head velocity:

(18) v0=J0θθ˙=−l1s1−l2s12−l2s12l1c1+l2c12l2c12θ˙1θ˙2.

*(5) Static Forces in the Human Model*. Forces and moments propagate from segment to segment. Torques on joints must be applied to keep the system in static equilibrium. Jacobian (*J*) in force domain maps force on the head into torques on joints:

(19) $$\begin{array}{c} \tau =J^{T}F, \end{array}$$

where *ℱ* is the Cartesian force required to act on the head.

**Figure 4:**
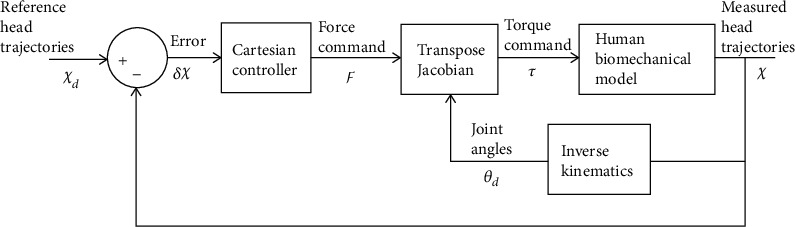
STS control scheme to emulate CNS.

*(6) Cartesian Control Law Design*. The control scheme is based upon the hypothesis that the feedback of head position *𝒳* to CNS, i.e., the STS controller, plays a role in carrying out STS motion. As shown in Figure 4, using the measured position of head *𝒳* and comparing with desired/reference head trajectories *𝒳*_*d*_, the CNS generates error signal *δ𝒳*. From head position measurements, the Estimator part of CNS infers joint positions (*θ*_*d*_), required to reduce the error *δ𝒳*. Similarly, the head position errors fed back to CNS generate torque command to the joints using the Cartesian control law. Since Cartesian control is usually implemented in force domain, the controller generates a force command *ℱ*. Then, the transpose Jacobian converts force command *ℱ* into torque command *τ*, for joint actuation.

## 4. Validation Framework

The human CNS modeling scheme to synthesize STS motion is designed in a purely analytical framework. To validate our modeling framework and the hypothesis that CNS control of STS motion has a dependence on head position trajectory feedback, we must check the model for its ability to replicate experimental STS motion using custom/subject-specific models. A comparison of simulations and experimental findings will be the basis of the validity of our control framework. The second phase of our study starts from scaling our analytical human model to custom models.

### 4.1. Subject's Physical Parameters and Anthropometric Conversion

Experimental data of sit-to-stand transfer were collected at Biomechanics Lab of Riphah International University. Seven healthy subjects (five males and two females, age: 22 ± 0.81 years, mass: 72.58 ± 11.61 kg, height: 1.70 ± 0.04 m) were selected for data collection of sit-to-stand motion. The subjects had no history of movement disorder. They provided their informed consent under the Ethics Committee of Riphah International University.

Subjects' physical parameter data (as shown in Table 2) are used to calculate BSP. An extensive literature is available on methods of anthropometric conversion. Among the various methods available in the literature [13], we have used the method of weighing coefficient [14], which is widely accepted among the research community. For brevity, only one representative data out of a total of 7 subjects is presented in Table 3.

### 4.2. STS Motion and Force Capture

Reference [24] provides comprehensive coverage of motion capture methods available. Of these, marker-based motion capture is termed as one of the most accurate methods. To measure ground reaction forces, a force platform has been used. These methods are extensively used in literature for modeling and analysis of biomechanical motion mechanisms [2, 15, 16]. For a detailed description of our experimental work, refer to [21]

#### 4.2.1. Experiment Protocol

**Figure 5:**
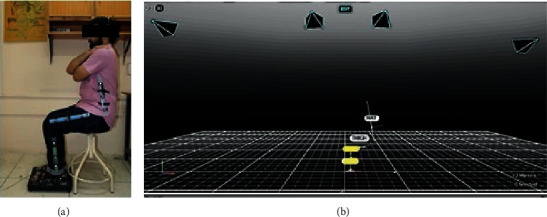
STS data capture setup: (a) a subject with markers affixed on segments. The feet are placed on force-plate, (b) motion capture view and cameras in Motive 2.0.1 environment.

Subjects completed the STS task using an armless chair 49 cm from the force-plate (as shown in Figure 5). To collect the data in the sagittal plane, three spherical reflective markers on the left side of each segment, i.e., foot, shank, thigh, and trunk, were attached. Since markers pose problems in the segment and joint position assessment due to skin or loose garment artifacts, a set of markers on each segment were applied using rigid rulers. One marker was attached on top of the head using a hairband. Motion capture was done using four infrared Flex 3 cameras by OptiTrack. The data were recorded at 100 Hz using the OptiTrack Motive 2.0.1 software. Force data were recorded at the same time using a 2-axis 4-beam Pasco force plate. The force data were captured at 100 Hz using the Capstone software.

Each subject completed multiple STS trials. All trials were done at once. Each trial began with the subject seated in the chair, arms crossed across the chest. The trial started with a verbal command of “stand,” and then, data were recorded for approximately 4 sec. After this, the subject was again asked to be seated and then trial repeated.

#### 4.2.2. Equipment and Calibration

To the best of our knowledge, this is the first study of STS motion capture in the sagittal plane (2D); hence, there are no definite rules available in the literature about the appropriate positions and number of markers placed on the body segments. Neither any research suggests an optimum number of cameras for reliable motion capture. Literature, in general, is about 3D motion capture [24, 25]. We have, therefore, opted for a multiple-camera system, along with spherical markers to ensure better visibility and reliable data reconstruction by the system. Cameras were arranged such that complete coverage of motion area could be ensured. Camera calibration using “calibration wand” and determination of frame of reference for motion capture area using “calibration square” were done before motion capture started. We used the 2-axis Pasco force platform for force data capture at 100 Hz in the Capstone data acquisition system. Before each trial, we checked the force plate for zero error.

#### 4.2.3. Data Collection and Analysis Tools

Each marker was manually numbered in the captured data file. Markers were then grouped into segments. Segment labels, too, were assigned manually in Motive Edit mode for each trial. Motive 2.0.1 generates motion capture data in *.tak* and *.c3d* file formats. For data analysis, we have used a motion capture software MoCap, a freely available motion data analysis toolbox that works seamlessly with Matlab. Force-plate data is collected from four-beam setup, that provides vertical and horizontal forces generated under both feet during STS. Force data is recoded in *.cap* format and exported into excel *.csv* format for analysis.

#### 4.2.4. MoCap Data Analysis

Motion data in *.c3d* format was imported into the Matlab MoCap toolbox for analysis. Marker positions were converted into joint positions. Then, angular positions of each joint in every frame were calculated. Similarly, the head position trajectory was constructed using a marker on the head. Marker data and joint data were used to animate the STS transfer of the subjects.

**Figure 6:**
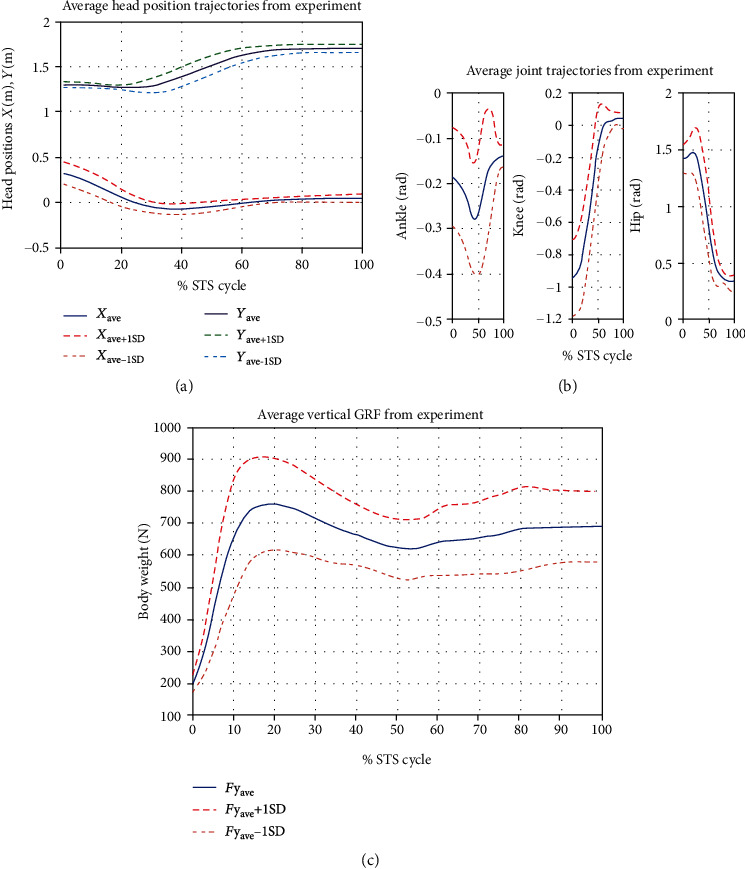
Ensemble average trajectories of (a) head position, (b) joint angles, and (c) GRF using motion capture. Curves in dashed lines represent ±1 standard deviation (SD).

Data on subject # 5 were corrupted and hence were rejected.  Figure 6(a) shows the ensemble average of head position; Figure 6(b) shows the ensemble average of ankle, knee, and hip joint trajectories; and Figure 6(c) shows the ensemble average of ground reaction force of all six subjects. Standard deviation curves in dashed lines show the magnitude of intrasubject variation.

### 4.3. STS Motion Control for Custom Models

We reconstruct STS motion using a custom human analytical STS controller framework. Subject-specific head position trajectories extracted from motion capture data are used as the reference.

## 5. Results

### 5.1. Simulations

**Figure 7:**
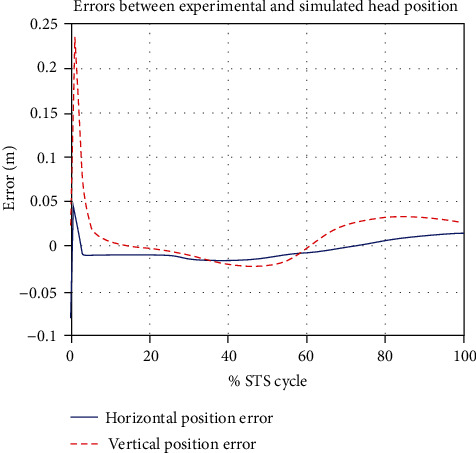
Comparison of ensemble average head position trajectories from motion capture experiments and simulations. RMS error for horizontal position *X* = 0.0118 m and for *Y* = 0.0315 m.

**Figure 8:**
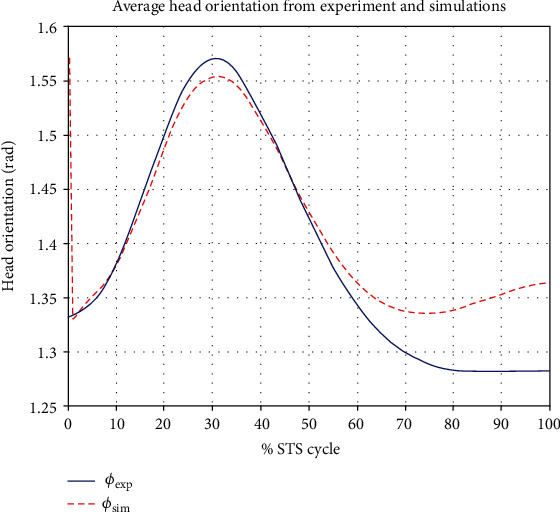
*RMS* *error* = 0.0442 *rad* for head orientation ∅, obtained from the average of experimental data and measurements from simulations.

The ensemble averages of all motion and force data obtained from the experiment and simulations are calculated and compared. The plots of the kinematic variable are shown in Figure 7 through Figure 8.

## 6. Discussion

In this study, we propose a modeling and motion control solution to evaluate the clinical hypothesis that besides numerous other factors, CNS controls the STS motion by tracking a prelearned head position trajectory. CNS compares this anticipated head motion pattern with actual head position measured by vestibular, proprioception, and vision senses. Based on the head position error, CNS generates torque commands for joints actuation so that a smooth STS motion may result. Motivated by the evidence for a task-oriented encoding of motion by the CNS [7], we present a human CNS modeling scheme to synthesize and control STS motion using an analytically generated head position trajectory in a high-level task control framework.

First, we realize a 4-segment 3-DoF analytical human biomechanical model based on anatomical proportions [9] in the sagittal plane. We realize the CNS model as an STS controller having two subsystems: an Estimator to automatically plan joint level motions and the Cartesian controller to generate appropriate joint torque commands to reduce head position error.

Our previous work [11, 18, 19] and some work from the literature [2, 9, 10, 20, 26] were based on the same analytical human model (realized in mathematical or simulation frameworks) using different combinations of measurements, feedbacks and controllers. We did the analytical design in the first phase to relate and compare our current study with the previous work. Using a well-defined human model and simulation results from previous studies helped us design and fine-tune the STS controller that could produce comparable results. As a standard procedure [8, 15, 16, 21], we validated our modeling and control scheme framework with laboratory data as well.

Physical parameters data of the 7 subjects (as shown in Table 2) are converted into BSP values using the weighing coefficient method of anthropometry. BSP values in Table 3 are used to scale customhuman models to match the anthropometry of the subjects.

**Figure 9:**
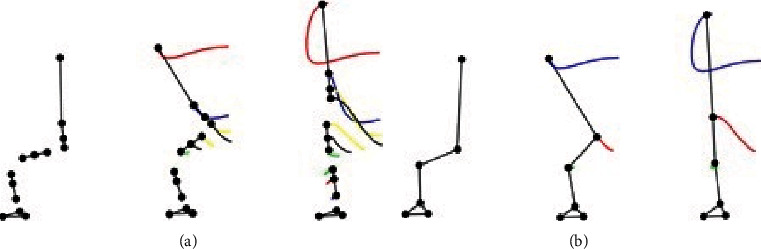
STS transfer phases with motion trajectories from animation based on (a) marker data and (b) joint data.

**Figure 10:**
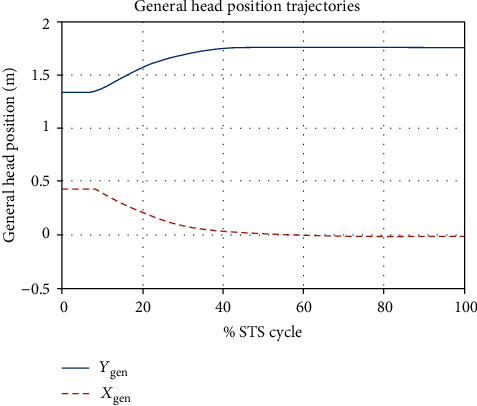
Analytically generated general head position trajectory.

We capture experimental kinematic data of STS motion in the sagittal plane using four OptiTrack Flex-3 cameras and thirteen spherical reflective markers on four segments of each subject. Kinetic data were collected at the same time using the Pasco force-platform underneath both feet of the subjects. The marker data were recorded in the OptiTrack Motive environment and then imported and analyzed using MoCap and MATLAB. The motion was reconstructed from marker data (as shown in Figure 9(a)). The animated motion helped check the data for missing markers and frames. The missing data were reconstructed using interpolation. The animation also helped determine the start and end of the STS cycle of all trials, and both the motion and force data were trimmed and normalized for % STS cycle. The marker data were then converted into six joints data (as shown in Figure 9(b)) which closely resembles the analytical model depicted in Figure 2. Experimentally generated head position trajectories in Figure 6(a) closely resemble the analytically generated general head position trajectory in Figure 10. The motion was then reconstructed in control and simulation framework by tracking the head marker trajectories in real time.

**Figure 11:**
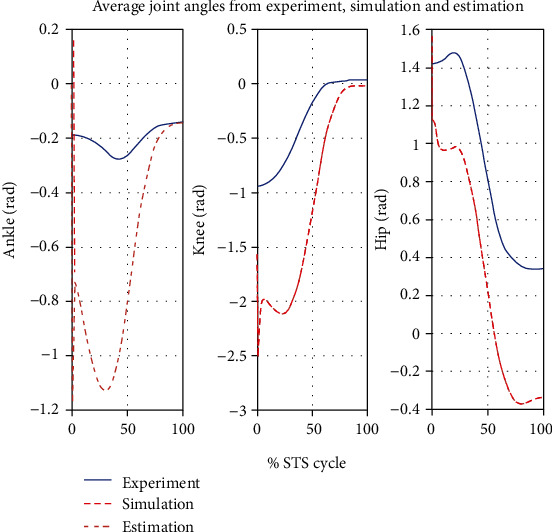
Comparison of average experimental joint trajectories with estimated and simulated trajectories. RMS error for ankle = 0.55 rad (estimation), 0.54 rad (simulation), for knee = 0.93 rad (both), and for hip = 0.59 rad (both).

**Figure 12:**
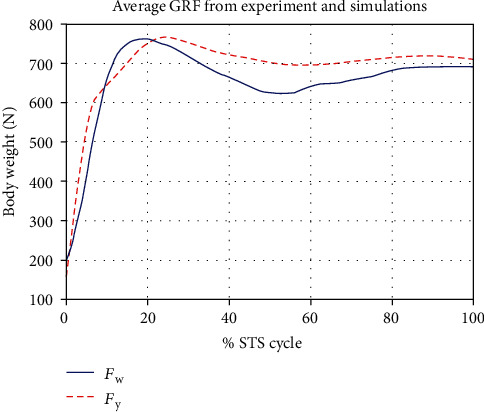
Average ground reaction force curve *F*_W_, measured by the force platform, showing the trajectory of body weight variation during STS by the subjects. *F*_y_ shows the same variable measured during subject-specific simulations. The RMS error between the two curves = 50.26 N.

**Figure 13:**
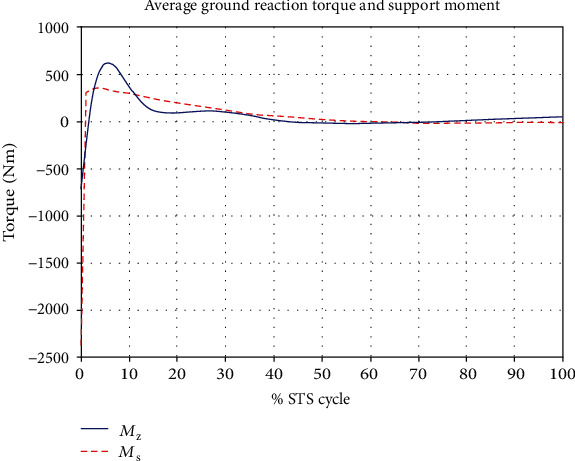
Average ground reaction torque *M*_z_ and support moment *M*_s_ (sum of joint torques). The two variables' correlation = 0.72.

**Figure 14:**
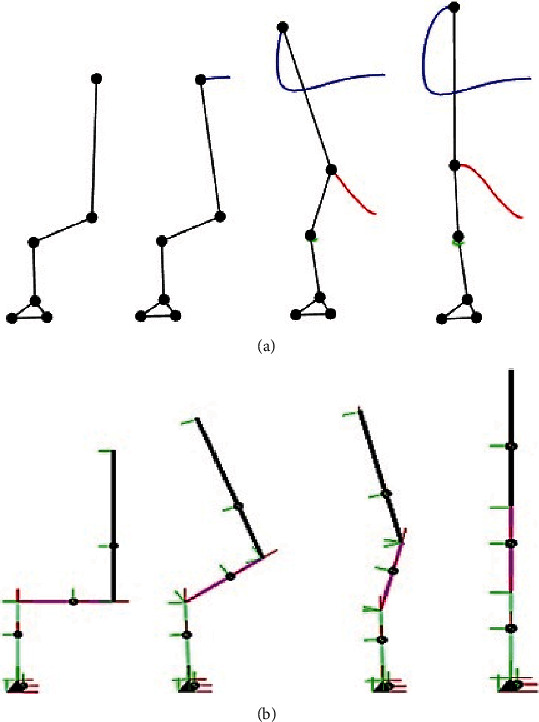
(a) Phases of STS from motion capture. Trajectories of joints are also shown. (b) Simulated STS motion in SimMechanics environment.

Figure 7 gives a comparison of experimental and simulated head position trajectories in horizontal (*X*) and vertical (*Y*) directions. The Cartesian control part of the STS controller provides appropriate joint torques to minimize head position error *δ𝒳*. The RMS error for *X* = 0.0118 m and for *Y* = 0.0315 m. This shows very good tracking of reference input *𝒳*_*d*_ by the STS controller. Experimental, estimated, and simulated joint angles are plotted in Figure 11. Estimated and simulated joint angles are compared with experimental joint angles. RMS error for ankle = 0.55 rad (estimation), 0.54 rad (simulation), for knee = 0.93 rad (both), and for hip = 0.59 rad (both). The joint angle errors are relatively high and attribute to the use of the same controller for a variety of custom human models and head position trajectories that exhibit relatively large intrasubject variations. The joint angle error can be reduced significantly if (1) the controller is tuned for each custom model and (2) simulation is run with subject-specific initial conditions. Another reason for larger joint angle errors attributes to the fact that the STS control strategy is based on head position tracking, and there are no joint position reference inputs and measurements being used. This is evident from small errors between experimental and simulated head position trajectories; Figure 8 plots head orientation curves ∅, measured from experiments and simulations. A small RMS error of 0.0442 rad for head orientation shows good estimation and tracking of the head trajectory by the controller. Kinetic variables are plotted and analyzed next.  Figure 12 shows how the force *F*_w_ exerted by the bodyweight during STS changes. At the start of the STS cycle, the initial force of 200 N shows the average weight of the two feet, shanks, and partially of thighs, while seated. With seat off, the weight on the force plate increases and so does the vertical component of the ground reaction force. The GRF measured from simulations is plotted as *F*_y_. The two forces match closely (RMS error 50.26 N only) and settle to the final value of the subject's average weight. Support moment *M*_S_ is the sum of ankle, knee, and hip joint torques. Ground reaction torque is a function of ankle joint torque [26]. We have found that a relatively high correlation (0.72) exists between ground reaction moment *M*_z_ and the support moment *M*_s_ as can be seen in Figure 13. The low RMS errors between experimental and simulated measurements validate our modeling framework.  Figure 14(a) depicts snaps from the animation of experimental STS.  Figure 14(b) shows STS motion phases from simulation, based on the customized human model in SimMechanics. The close resemblance between the animation of experimental data and simulation shows the good quality of STS motion control which attributes to (1) robust design of analytically developed STS controller to model CNS, (2) reliability of experimental data capture techniques employed, and (3) low error factor in BSP conversion from weighing coefficient method to obtain customized human biomechanical models.

### 6.1. Assumption and Limitations

The subjects' physical parameters were converted into complete set of BSP using weighing coefficient method, which is a mathematical method of anthropometery. Despite the risk of high error in estimation [13], this method is widely accepted in research community due to its convenience as compared to other methods that need special equipment for body segment measurements. The estimation error, however, leads to modeling error that becomes a source of mismatch in experimental and simulation results. Moreover, there is a lack of protocols for motion capture in 2D. We devised a set of protocols for this experiment which we kept modifying until a satisfactory level of reliable results was achieved. There were some limitations associated with experimental equipment as well: (1) we did not have specialized skin tight garments for subjects. Since markers pose problems in the segment and joint position assessment due to skin or loose garment artifacts, a set of markers on each segment were applied using rigid rulers. (2) The motion capture equipment and force plate were not synchronized electronically; the two variables were visually analyzed from captured data for time synchronization. Another assumption was made by using same motion controller for all subject specific human biomechanical models. Further improvement in work could be made if controller were tuned separately for each scaled model. For this study, our modeling scheme was based on rigid body segments; such assumption leads to modeling error of the systems like human body that are not exactly rigid.

## 7. Conclusion

A modeling framework to evaluate the role of head position trajectory in physiologically relevant STS motion control by the CNS is presented. A robotic approach for the synthesis of STS motion using task-level control is utilized. We mapped a scaled dynamic human model to the human subjects' anthropometric values and simulated STS motion by tracking head position trajectories in real time. The study contributes to the knowledge base by proposing a system that (1) synthesizes human motion using a high-level task control framework, for which low-level motion control is automatically generated and (2) validates a 2D biomechanical modeling scheme based on the weighing coefficient method for inference of Body Segment Parameter (BSP). The modeling scheme is validated using kinematic and kinetic analyses of simulated and captured motion and force data of real subjects. The analytically designed STS controller is robust enough to simulate real subjects' STS motion. Low errors between experimental and simulated motions not only prove the validity of the modeling framework but support the clinical hypothesis that there exists a role of head position measurement feedback to CNS in controlling a smooth STS motion.

In the future, we want to modify the human biomechanical modeling scheme from rigid body kinematics to account for elastic body links to better match subject-specific anthropometry. Our hypothesis and findings can be further generalized to all kinds of human motion syntheses like walking and stair climbing.

## Figures and Tables

**Table 1:** BSP data for analytical biomechanical model [9].

	Segment	Mass (kg)	Length/height (m)	Center of gravity (m)	Moment of inertia (kg m^2^)
General human model	Foots	1.91	0.07	—	—
	Shanks	6.14	0.43	0.25	0.11
	Thighs	13.20	0.43	0.25	0.26
	HAT	44.75	0.83	0.31	7.53

**Table 2:** Subjects' physical parameter data.

Subject ID	Gender	Age (year)	Mass (kg)	Height (m)
1	Male	21	76.55	1.69
2	Male	22	79.81	1.70
3	Male	21	50.05	1.69
4	Female	22	66.56	1.61
5	Female	22	84.91	1.67
6	Male	23	71.05	1.72
7	Male	23	79.10	1.78

**Table 3:** BSP data based on the subject's physical parameters.

Subj ID	Segment	Mass (kg)	Length/height (m)	Center of gravity (m)	Moment of inertia (kg m^2^)
1	Foots	2.22	0.066	—	—
	Shanks	7.11	0.419	0.237	0.114
	Thighs	15.31	0.417	0.236	0.278
	HAT	51.90	0.801	0.299	8.199

## Data Availability

Readers can request the corresponding author for motion and force capture datasets.

## References

[1] van Lummel R. C. (2017). *Assessing sit-to-stand for clinical use*.

[2] Mughal A. M., Iqbal K. (2019). Optimization of biomechanical STS movement with linear matrix inequalities. *International Journal of Mechatronics Systems and Control*.

[3] Chiba R., Takakusaki K., Ota J., Yozu A., Haga N. (2016). Human upright posture control models based on multisensory inputs; in fast and slow dynamics. *Neuroscience research*.

[4] Forbes P. A., Siegmund G. P., Schouten A. C., Blouin J.-S Ã.©b. (2015). Task, muscle and frequency-dependent vestibular control of posture. *Frontiers in Integrative Neuroscience*.

[5] Siriphorn A., Chamonchant D., Boonyong S. (2015). The effects of vision on sit-to-stand movement. *Journal of Physical Therapy Science*.

[6] Scholz J. P., Reisman D., Schöner G. (2001). Effects of varying task constraints on solutions to joint coordination in a sit-to-stand task. *Experimental Brain Research*.

[7] Vincent D. S., Chen R. (2012). A task-level biomechanical framework for motion analysis and control synthesis. *Human Musculoskeletal Biomechanics*.

[8] Khatib O., Demircan E., de Sapio V., Sentis L., Besier T., Delp S. (2009). Robotics-based synthesis of human motion. *Journal of Physiology-Paris*.

[9] Iqbal K., Pai Y. C. (2000). Predicted region of stability for balance recovery: motion at the knee joint can improve termination of forward movement. *Journal of Biomechanics*.

[10] Mahmood M. A., Iqbal K. (2012). Physiological LQR design for postural control coordination of sit-to-stand movement. *Cognitive Computation*.

[11] Rafique S., Najam-ul-Islam M., Mahmood A. (2019). Sit-to-stand motion control using head position feedback to CNS. *Basic & Clinical Pharmacology & Toxicology. Vol. 124*.

[12] Geravand M., Korondi P. Z., Werner C., Hauer K., Peer A. (2017). Human sit-to-stand transfer modeling towards intuitive and biologically-inspired robot assistance. *Autonomous Robots*.

[13] Riemer R., Hsiao-Wecksler E. T. (2009). Improving net joint torque calculations through a two-step optimization method for estimating body segment parameters. *Journal of biomechanical engineering*.

[14] Winter D. A. (2009). *Biomechanics and motor control of human movement*.

[15] Caruthers E. J., Thompson J. A., Chaudhari A. M. W. (2016). Muscle forces and their contributions to vertical and horizontal acceleration of the center of mass during sit-to-stand transfer in young, healthy adults. *Journal of Applied Biomechanics*.

[16] Cullen M. K. (2015). *Muscle-driven simulations of sit to stand transfer in persons with severe osteoarthritis, [M.S. thesis]*.

[17] Campos Padilla I. Y. (2016). *Biomechanical analysis of the sit-to-stand transition [Ph.D. thesis]*.

[18] Rafique S., Najam-l-Islam M., Mahmood A., Benavente-Peces C., Slama S., Zafar B. (2019). Synthesis of sit-to-stand movement using SimMechanics. *Proceedings of the 1st International Conference on Smart Innovation, Ergonomics and Applied Human Factors (SEAHF). SEAHF 2019. Smart Innovation, Systems and Technologies, vol 150*.

[19] Rafique S., Mahmood A., Najam-ul-Islam M., Arai K., Bhatia R., Kapoor S. (2019). Robust control of physiologically relevant sit-to-stand motion using reduced order measurements. *Proceedings of the Future Technologies Conference (FTC) 2018. FTC 2018. Advances in Intelligent Systems and Computing, vol 881*.

[20] Mughal M., Iqbal K. (2005). A fuzzy biomechanical model for H_∞_ suboptimal control of sit-to-stand movement. *International IASTED Conference on Intelligent Systems and Control*.

[21] Rafique S., Islam M. N., Shafique M. Position driven sit-to-stand simulation using human body motion and force capture.

[22] Mughal A. M., Iqbal K. Synthesis of angular profiles for bipedal sit-to-stand movement.

[23] Craig J. J. (2005). *Introduction to robotics: mechanics and control*.

[24] van der Kruk E., Reijne M. M. (2018). Accuracy of human motion capture systems for sport applications; state-of-the-art review. *European Journal of Sport Science*.

[25] Bilesan A., Owlia M., Behzadipour S. (2018). Marker-based motion tracking using Microsoft Kinect. *IFAC-PapersOnLine*.

[26] Mughal A. M., Iqbal K. (2010). Experimental analysis of kinetic variables for biomechanical sit to stand movement. *34th Annual Meeting of American Society of Biomechanics*.

