# Corrigendum to “Cartesian Control of Sit-to-Stand Motion Using Head Position Feedback”

**DOI:** 10.1155/2023/9798458

**Published:** 2023-10-12

**Authors:** Samina Rafique, M. Najam-ul-Islam, M. Shafique, A. Mahmood

**Affiliations:** ^1^Electrical Engineering Department, Bahria University, Islamabad 44230, Pakistan; ^2^Biomedical Engineering Department, Riphah International University, Islamabad 44230, Pakistan

In the article titled “Cartesian Control of Sit-to-Stand Motion Using Head Position Feedback” [1], similarity with the methodology used and an amount of text overlap has been identified with the authors' previously published article:

Rafique S, Najam-ul-Islam M, Shafique M, Mahmood A. Neuro-fuzzy control of sit-to-stand motion using head position tracking. *Measurement and Control*. 2020;53(7-8):1342–1353. doi:10.1177/0020294020938079 [2].

The authors apologise for not making this clear in the article and wish to acknowledge this related study.
